# Developing the Creative Communities Framework for Living Well With Serious Mental Illness: Findings From a Realist Evaluation

**DOI:** 10.1002/jcop.70028

**Published:** 2025-07-10

**Authors:** Louisa Anne Smyllie‐Peters, Tim Gomersall, Mike Lucock

**Affiliations:** ^1^ University of Huddersfield Huddersfield UK; ^2^ Leeds Trinity University Leeds UK

**Keywords:** community, creativity, realist evaluation, recovery, serious mental illness

## Abstract

To answer the research question how, why and in what context do community arts organisations establish a safe and empowering space that allows individuals with serious mental illness to engage with recovery processes. A realist evaluation was conducted between 2021 and 2023. This paper presents findings from primary data collection utilising arts elicitation interviews with 12 participants with serious mental illness at 2 time points. A further four realist interviews were conducted with workers from community organisations. Template analysis was the main analytical tool used to test and refine an initial programme theory, incorporating sub‐analyses of visual and longitudinal data. Theory was developed that explains how the interactions between the lived experiences and community interventions creates a safe and empowering space. Six key ingredients were identified including (1) community setting; (2) creative activity; (3) consistent access; (4) choice over engagement; (5) shared lived experiences; (6) compassionate workers; forming the Creative Communities Framework. Creative communities present an alternative context to clinical spaces, to support individuals to live well with serious mental illness.

## 1 Introduction

Gaps within recovery provision for serious mental illness (SMI) have been identified across Western countries such as the UK, Australia and USA, which relate to increasing demands that service providers are not able to meet due to funding and resource limitations (Darzi 2024; House of Commons Committee 2023; Petrie et al. 2021; Reinert et al.^ 2024^). Social determinants of health also impact the extent to which health services can address increasing demands, resulting in a “postcode lottery”, that is inconsistent access to care due to arbitrary factors such as the area in which you live (Capehorn et al. 2016; Frost et al. 2019; Kaul et al. 2021; Marmot 2010; Stevens et al. 2015). The gap in service provision is particularly pertinent within SMI care, which has been identified as a key public health issue due to increasing rates globally, poor health outcomes, and static recovery rates (NHS 2016; Reinert et al. 2024; Whiteford et al. 2013). In a recent review of healthcare services in the UK, clinical spaces for SMI care were found to vary widely, with increases in restrictive interventions and premature deaths (Darzi 2024). Recent healthcare policy acknowledges that nonclinical, community‐based interventions are helping to address gaps in care provision (NHS England 2019) by supporting “traditional” healthcare provision. One such intervention is that of community arts activities, with recent research commissioned by the World Health Organisation demonstrating that engaging with arts‐based activities can help manage mental illness (Fancourt and Finn 2019). However, there is a call for more research to provide explanatory insights into how and why recovery can occur within specific contexts such as community arts, especially for individuals living with SMI (Fancourt et al. 2021; Leamy et al. 2011; van Weeghel et al. 2019). A systematic realist review aimed to develop new theories to explain how, why and in what context community arts enables identity change as part of a recovery process and proposed an initial programme theory which suggested that people with SMI found valuable social and psychological resources within community‐based arts activities (Peters et al. 2023). The review findings revealed that a safe and empowering intervention context was vital to activating mechanisms of change (such as acceptance), that lead to the outcome of engaging recovery processes within community arts (Peters et al. 2023). These findings raised new questions about how this intervention space is established at an organisational level, and what psychological processes are involved in developing a feeling of safety at an individual level. The current paper builds on the findings of the realist review and initial programme theory by presenting findings from the next stage of a realist evaluation where primary data was collected (through qualitative interviews) to determine the context and mechanisms that lead to the outcome of a safe and empowering space within community arts.

Within the current paper, a personal recovery definition is used, characterised as an individual, nonlinear “process whereby people with [serious] mental illness progress to live autonomous, contributing and satisfying lives in the community, even with persisting symptoms” (Whitley et al. 2015, 951). The empirically established recovery processes of connectedness; hope; identity; meaning; empowerment; and difficulties were used as a starting point to help navigate the complexity of SMI recovery (Bird et al. 2014; Leamy et al. 2011; Stuart et al. 2017; Slade et al. 2014; van Weeghel et al. 2019). These terms reflect a broad and flexible view of recovery and were used as “sensitising concepts” (Bowen 2006) within the current research to give structure and form to our analysis. There is evidence that community based social interventions can support recovery for individuals living with SMI but there is a great deal of variation in the types of community settings (Killaspy et al. 2022). Community is understood within this study as a fluid space formed by the social and psychological features of a particular group of people, within a shared physical environment (Lazarus et al. 2017; Pahwa et al. 2021; Townley 2015; Walker et al. 2017). Evidence exists that community spaces, which encompass charitable organisations and community centres in which activities take place, can engage recovery processes for individuals living with SMI. Tucker (2010) found that community spaces empower individuals by allowing them to assert a level of control to shape the physical and social environment, which is not possible within healthcare settings. Walker et al. (2017) found that informal community spaces develop ‘meaning’ through an “environment where people encounter a range of resources, activities, experiences and relations without assuming the identity of a person receiving therapy” (pg. 52). The Social Identity Approach to Health (Haslam et al. 2018) highlights how the ‘identity’ recovery process is engaged through a sense of group belonging. For example, Best et al. (2016) found that individuals with addictions were able to maintain sobriety when they identified with other people attending alcoholics anonymous groups, as the group promoted a ‘recovery’ identity. However, such research illustrates that community spaces are comprised of numerous contextual features, which in turn can engage multiple recovery processes. As such there is a need to identify the contextual elements within specific community spaces to understand how and why they engage personal recovery processes (Fancourt et al. 2021).

One such community space is that of community arts. The arts is a broad term that often refers to a type of creative expression and encompasses a multitude of different art practices including (but not limited to) drama, film, music, literature, painting, drawing, sculpture and crafts (Azmat et al. 2015; Belfiore and Bennett 2007; Davies 2008). The term community arts does not refer to a specific type of art, but rather the art practices and activities used by people who identify as part of a particular community (Mulligan et al. 2008; Tate 2023). Recent research that has explored community activities more broadly has identified hundreds of potential outcomes and psychosocial mechanisms, which are activated in certain contexts (Fancourt et al. 2021; Polley et al. 2020). Such findings illustrate the complex and contextually based nature of SMI recovery within community arts, which makes it difficult to determine what works, for whom and in what context. Therefore, the present study aimed to understand how, why and in what context do community arts organisations establish a safe and empowering space that allows individuals to engage with recovery processes? This will highlight the mechanisms of change within the intervention space and contextual features that activate these mechanisms.

## 2 Materials and Methods

**Figure 1:**
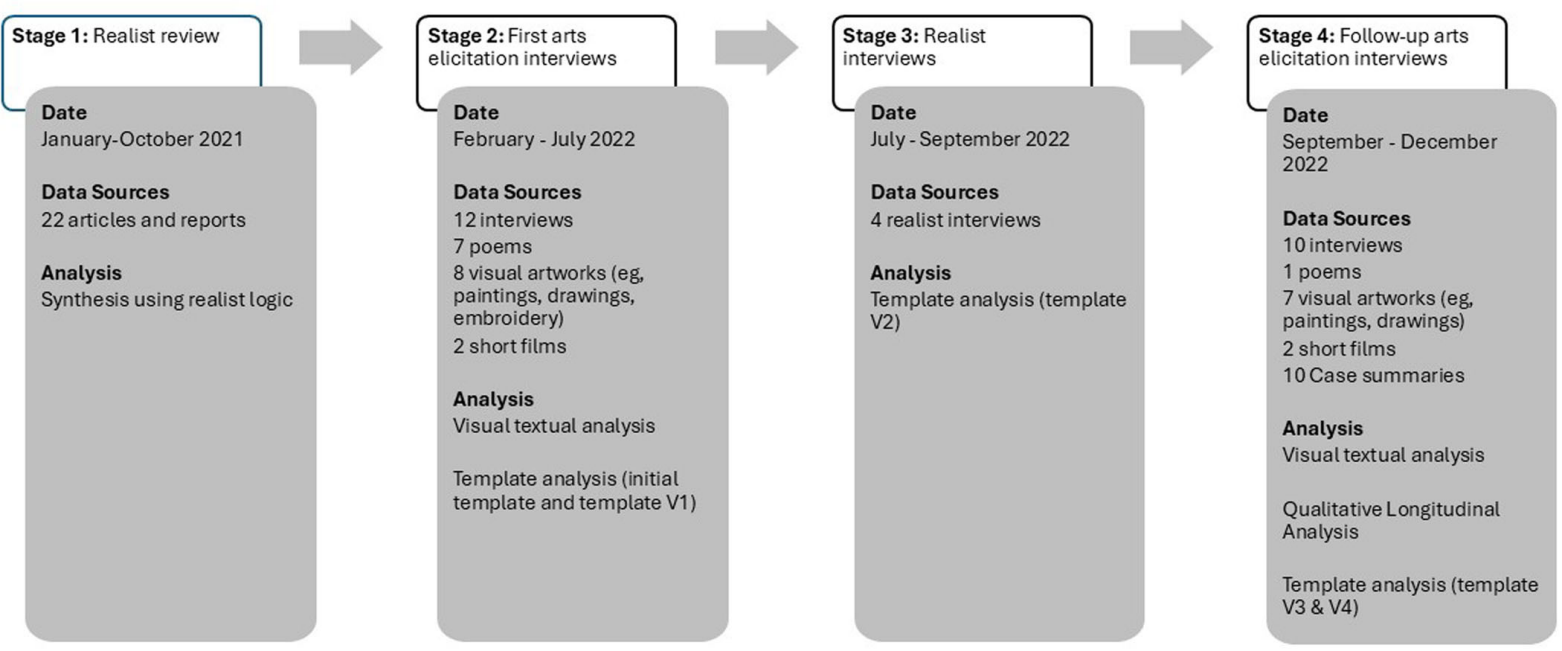
A flow diagram outlining the four stages of the realist evaluation process, including data collection dates, data sources and analysis.

Figure 1 shows the research process which included the realist systematic literature review as phase 1 (Peters et al. 2023) which developed the initial programme theory and stages 2–4, reported in this paper, in which the initial programme theory was tested and refined, The findings from stages 2–4 of the evaluation therefore builds upon the findings of the realist systematic review, and offers an analysis of the context, followed by a deeper focus on the mechanisms that can be activated within this. This realist evaluation was conducted between January 2021 and December 2022 using the approach outlined by Pawson and Tilley (1997) which is an effective approach to evaluate complex social and health interventions (Skivington et al. 2021; Wong 2018). Realist research is an iterative, theory‐driven approach to evaluating or synthesising evidence from multiple sources to develop evidence‐informed theory about how an intervention works (i.e., a programme theory) (Jagosh et al. 2014; Pawson 2006; Pawson and Tilley 1997). Ethical approval for stages 2–4 of the realist evaluation was gained from the University of Huddersfield, UK (Ref: SREIC/2021/117) in February 2022.

### 2.1 Participants

Two participant groups were recruited using purposive sampling including individuals living with SMI and workers within community organisations. Inclusion criteria for individuals living with SMI included adults aged 18 or over; currently attends a community art for mental health group; and living with a serious mental illness, that is a mental health condition that severely impairs daily functioning, for example schizophrenia, bipolar disorder, or long‐term depression. It was not a requirement for participants to have a formal diagnosis or ‘prove’ their illness. Recruitment was conducted in partnership with four organisations across three local authority areas in the UK. The recruitment criteria for workers included adults aged 18 or over and working (either paid or voluntary) at a community organisation that offers arts activities for individuals living with SMI. The recruitment criteria for both groups of participants were purposefully broad to ensure a diverse range of participants. A heterogeneous sample is desirable within realist research to ensure a phenomenon is explored from multiple perspectives, which aids theory development (Pawson 2006).

### 2.2 Arts Elicitation Interviews

Arts elicitation interviews are an adaptation of the semi‐structured photo elicitation interview outlined by Bates et al. (2017). Two interviews were conducted within a 6‐month time period to explore change over time. Participants were asked to bring their own artworks to the interviews in response to the prompt ‘what does your mental health condition mean to you’ (interview 1) and ‘your experience of attending your art group’ (interview 2). The artworks were used as both discussion prompts in the interview, and as objects of data in their own right. If a participant did not bring any artworks to the interview, a photo image pack was used, which was created by the researcher. There were two image packs of approximately 30 images for each interview. The photos in the first interview were focused on the lived experience of SMI and the photos in the second interview were focused on potential recovery processes. Image packs were created through online searches using terms drawn from the findings of the systematic review in stage 1 of the realist evaluation (Peters 2023), such as ‘isolation’ and ‘identity change.’ These searches were filtered to open access online sources only, to ensure selected images could be used for research purposes (King et al. 2019). The images selected were art‐based, that is, photos of paintings, rather than explicit imagery or literal representations of illness. These images were used as discussion prompts only and not included in the analysis process.

Creative methods, such as photo elicitation interviews, have been found to be appropriate for exploring sensitive topics such as SMI, by providing an alternative, non‐verbal communication tool (Calman et al. 2013; Collier and Collier 1986; Glaw et al. 2017; King et al. 2019; Vansteenkiste et al. 2020). Furthermore, using arts‐based prompts draws on shared social knowledge helping to break down communication limitations between the researcher and participant, and promote self‐expression, which can be a particular difficultly for individuals with SMI (Glaw et al. 2017; Harper 2002; Milasan et al. 2020; Pain 2012).

### 2.3 Realist Interviews

Realist interviews were conducted with four workers from partner organisations to provide another perspective on community arts and recovery. Interviews with workers aimed to provide a different perspective on the developing theories and so an alternative technique was needed. A realist interview is a type of semi‐structured interview that is designed to explicitly validate, falsify, and modify theory through stakeholder interpretations (Manzano 2016). This is achieved through an interchangeable teacher‐learner dynamic between the researcher and participant, whereby the researcher presents developing theories and becomes the learner as the participant discusses the theories drawing on their on‐the‐ground knowledge (Manzano 2016; Pawson and Tilley 1997).

### 2.4 Analysis

Template analysis was used as the main analysis tool within this evaluation as it can be applied within a range of epistemological approaches and aims to balance a structured analytical approach with flexibility (Brooks et al. 2015; King and Brooks 2017). The iterative development of templates throughout the analysis aligns with the ‘test and refine’ approach used within a realist evaluation. Furthermore, template analysis enables the use of a priori themes (King and Brooks 2017), which allows for a theory‐driven realist analysis (Pawson and Tilley 1997). Coding used involved identifying and labelling different interacting elements within the intervention, which included specific contexts (C), mechanisms (M), and outcomes (O). The interaction between these elements, articulated as CMO configurations, present causal explanations and are used to develop programme theory (Pawson 2006; Pawson et al. 2005; Wong et al. 2013). Therefore, the templates reflected the evolving programme theories, with each template providing an audit trail of theory development to improve transparency and quality checking (King and Brooks 2017). A copy of the templates can be found within the Supporting Information. Further quality checks were carried our during the analysis to improve trustworthiness and rigour, including all members of the research team applying the initial template to a selection of the data (King and Brooks 2017). Codes were compared and discussed and there was general agreement that the template being developed reflected the data, illustrating Inter‐researcher reliability.

The data collection methods generated visual, textual, and longitudinal data for participants with SMI, therefore, sub‐analyses were conducted before applying the template to the data. A multi‐analytical approach is also recommended when conducting longitudinal qualitative research to explore data both cross‐sectionally and over time (Calman et al. 2013; Hermanowicz 2013; Lewis 2007). Arts‐based data were examined using systematic visuo‐textual analysis to produce textual interpretations of art‐based data (Brown and Collins 2021). A systematic visuo‐textual analysis involves three phases of analysis whereby the visual and textual datasets are analysed separately, and then interpretations are combined. First the artistic/linguistic elements of the visual artefact and transcript are described, reflecting on features such as structure, focus, action, colour, words, phrases, and content etc (Brown and Collins 2021). The second phase involves conceptualising the data through interpretation, drawing out key elements and patterns that offer meaning in relation to the topic under review (Brown and Collins 2021). Lastly connections between the visual and textual interpretations are drawn to develop themes. This approach also tempers researcher interpretations by combining data with interview data to ensure participant perspectives are captured and inform the overall interpretation of the artwork. A Qualitative Longitudinal Analysis (QLA) was used to explore both change and processes taking place over time (Hermanowicz 2013). QLA involved reviewing the first and second interviews for each participant using specific questions such as what changed over time and in what way? (Calman et al. 2013; Lewis 2007; Saldaña 2003). The notes from the QLA were then used to create ideographic case summaries, to which the template was applied (Hermanowicz 2013; Lewis 2007). An example from each of these sub‐analyses can be found within the Supporting Information. The results and inferences drawn from the sub‐analyses were incorporated into the template analysis to inform theory development.

## 3 Results

A total of 16 participants were recruited for the study through 4 partner organisations, 12 living with SMI and 4 workers. A total of twenty‐six interviews were undertaken, with of the participants with SMI being interviewed twice. Full details of participant demographics can be found in Table 1.

**Table 1:** Participant demographic information for both individuals living with SMI and workers.

Arts elicitation interview participants	
Age	24–75
Gender	Female (4); Male (8)
Ethnicity	White British (8)
	Black British (1)
	British Irish (1)
	Latina (1)
	British of European/South Asian descent (1)
Length of SMI	8–55 years
Disclosed SMI	Depression, anxiety, PTSD, eating disorder, addiction, OCD, psychosis, self‐harm, suicide ideation, learning disability
No. of other conditions reported including	Acquired brain injury (1); autism (2); carpel tunnel (1); chronic obstructive pulmonary disease (1); chronic pain (3); diabetes (1); epilepsy (1); fibromyalgia (1); gallstones (1); heart condition (1); migraines (1); mobility disability (2); respiratory issues (1); sleep aponia (1); underactive thyroid (1)
Interviews undertaken Follow‐up interval	Initial interviews (12)
	Follow‐ups (10)
	6 months (9)
	3.5 months (1)
Realist Interview Participants	
Gender	Male (1); Female (2); nonbinary (1)
Ethnicity	White British (4)
Lived experience of SMI	Yes (1)
	Unknown (3)
Job role	Facilitator and coordinator (3)
	Coordinator (1)

**Figure 2:**
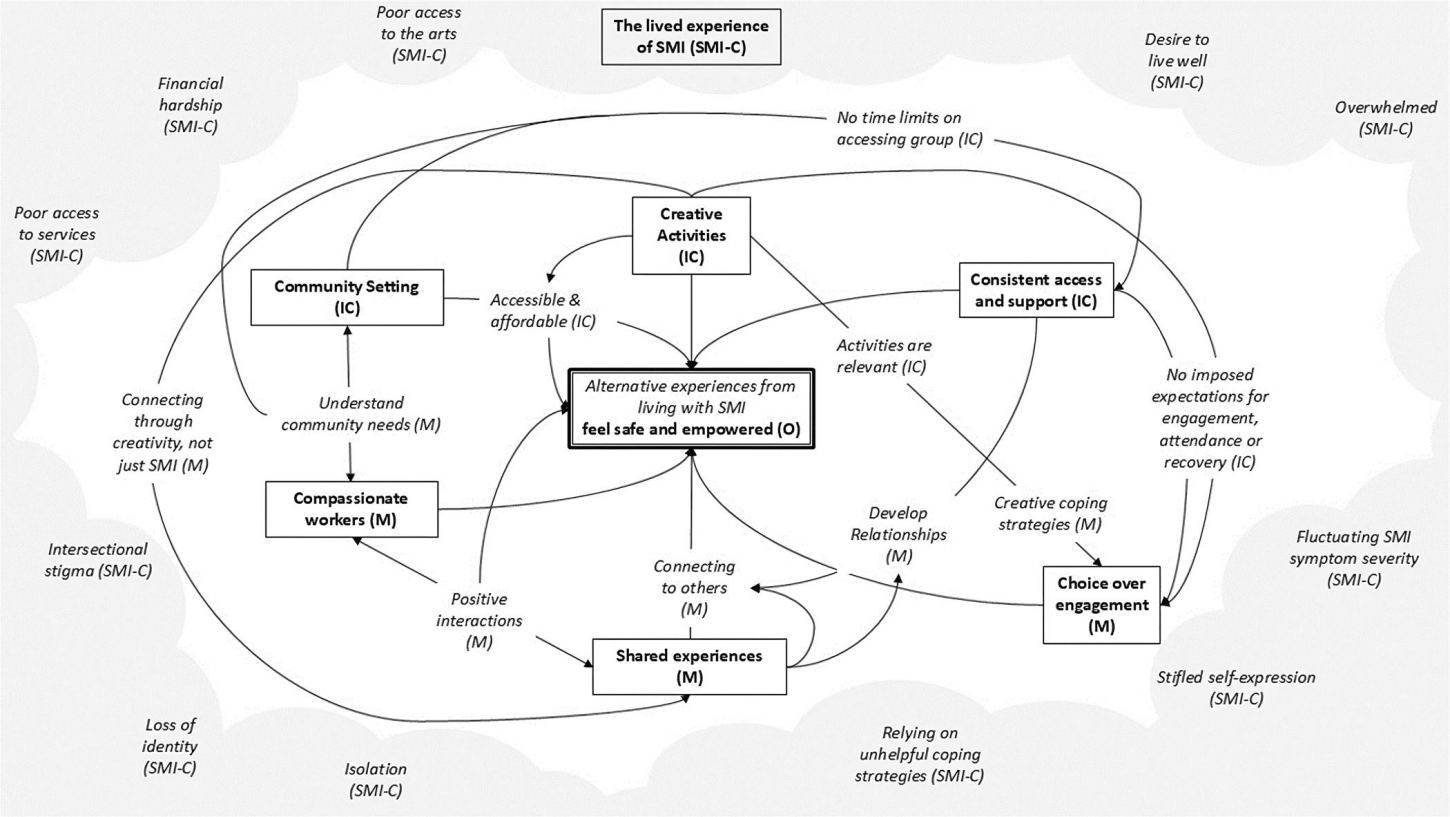
CMO configurations identified within the programme theory. SMI‐C refers to the contextual features of living with SMI; IC refers to the Intervention Context; M refers to mechanisms; O refers for the outcome. Details highlighted in bold with a border indicate key CMO elements, which have been numbered to reflect the written programme theory, with details of these provided in italics.

The primary data developed a new programme theory to answer the question, how, why and in what context do community arts organisations establish a safe and empowering space that allows individuals to engage with recovery processes? The programme theory was developed from several interrelated CMO configurations illustrated in Figure 2.

Figure 2 illustrates the dynamic and complex interactions between the intervention contextual features and the mechanisms activated. Six key ingredients were identified (three intervention contextual features and three mechanisms) that are needed to establish a safe and empowering creative community in which individuals with SMI can engage with recovery processes (the outcome). These contexts and mechanisms are identified in Figure 2 (highlighted in bold with a border) by the multiple interactions across several CMO configurations. Notably, these processes operate within the context of living with SMI, indicated by the contextual features around the edge of the diagram in grey. The context of living with SMI was also found to be important to understand the activation of mechanisms. The programme theory develop from this study is centred around these key CMO's and is outlined in full below:

**Programme Theory: Establishing a safe and empowering creative community**

If the lived experience of SMI is challenging and exacerbated by social issues including stigma and deprivation (context of SMI), then creative activities (intervention context 1) organised by compassionate workers (intervention context 2) in a community setting (intervention context 3) offers connection to others through shared experiences (mechanism 1), choice over engagement (mechanism 2), and consistent access to a community (mechanism 3). This develops a safe and empowering creative community space in which alternative experiences can occur (outcomes).

The six ingredients (intervention contexts and mechanisms) where used to construct the Creative Communities Framework as key features of the intervention. The Creative Communities Framework is presented in further detail below and outlined in Figure 6. However, these ingredients operate within the lived experiences of individuals with SMI, which is often a negative experience and involves factors a both the individual level (loss off identity) to wider social issues (intersectional stigma). As such the context of the SMI lived experience first needs to be outlined to fully understand the interactions between the SMI lived experience and framework ingredients.

### 3.1 Context of the SMI Lived Experience

The lived experience of SMI was found to be challenging, relating to individual experiences such as symptom severity, which are exacerbated by the macro level social determinants of health of stigma and deprivation. As a result, individuals with SMI experience a lack of safe spaces within both mental health services and their day‐to‐day life, which hinders engagement with personal recovery processes. At an individual level, SMI can pose daily challenges of coping with symptoms that fluctuate in severity, exemplified by George's experience of PTSD.

“When I have nightmares, [it] takes me straight back to the accident. I would get all the smells, I still hear all the screaming and shouting. I still remember it. Hearing someone shouting out, ‘are they dead, are they dead?”

(George, first interview)

**Figure 3:**
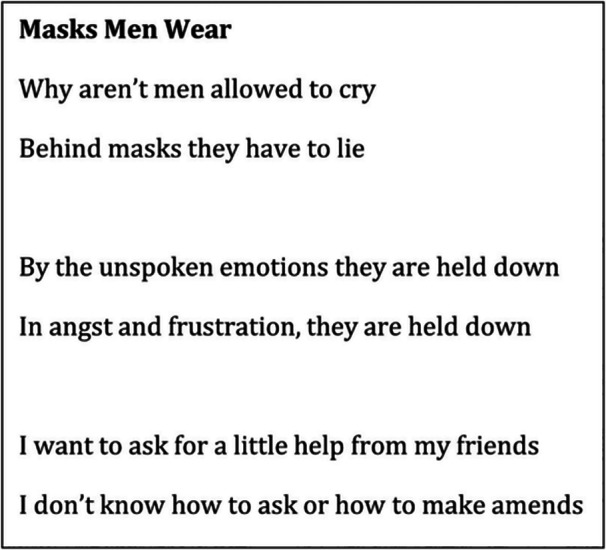
An excerpt from the Poem ‘Masks Men Wear’ by Rob.

Regardless of diagnosis, participants all commented on feeling overwhelmed by SMI; “I'm numb and it is all raging around me… I can't keep up and I don't want to” (Viv, first interview); “your illness is part of every waking moment” (Alex, realist interview). These overwhelming experiences lead to a loss of identity, “When I went through me relapse about 3 years ago, my identity was shattered again” (Cam, first interview). These comments highlight the reality of SMI as a chronic condition and the significant impact on day‐to‐day life. The constant need to manage symptom severity often led to a reliance on unhelpful coping strategies including masking and addiction. Masking relates to hiding parts of self as a response to distress and stigma in relation to SMI, as depicted by Rob's poem in Figure 3.

The poem illustrates how masking can isolate a person from their support networks (‘friends’) and stifle self‐expression of emotions such as ‘rage’. Whilst masking can offer some initial respite, it is not a sustainable state, as one participant describes:

“when I got unwell, I wasn't able to function with any of those masks anymore. … after a while, the mask just wore thin because it wasn't ‐ I wasn't presenting how I wanted… I wasn't able to function… so I'd mediate that by wearing a mask and… also heavy drinking”.

(Sam, first interview)

Both Rob and Sam's experience highlight how stifling self‐expression and masking can lead to the use of other unhelpful coping strategies such as self‐harm and addiction. However, these experiences are not solely a result of SMI symptoms, but rather wider social issues including deprivation and intersectional stigma relating to multiple parts of identity.

**Figure 4:**
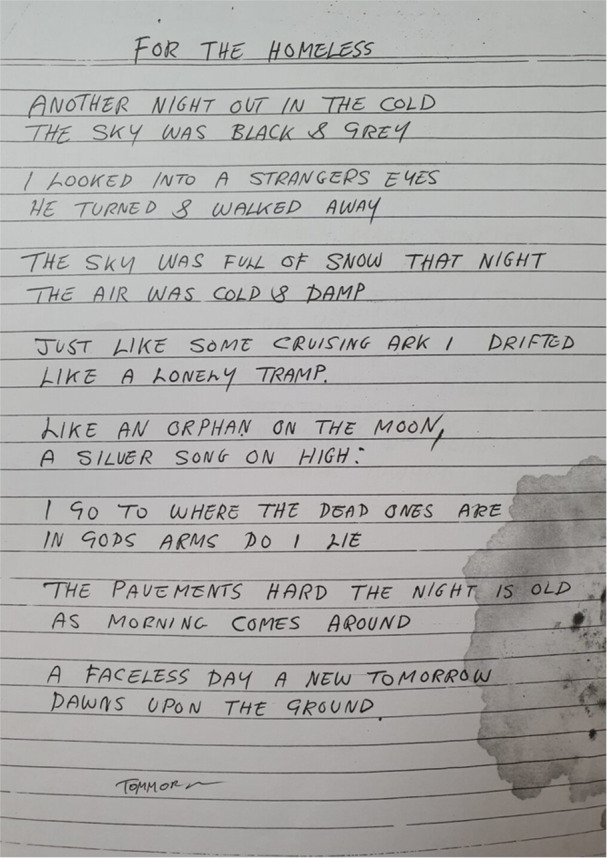
An excerpt from the Poem ‘For the Homeless’ by Stan.

Experiences of deprivation were reported by participants in relation to food poverty: “I wasn't eating properly as well because I haven't got money to buy stuff that I should be eating” (Kyle, first interview), and uncertain housing as exemplified by the below poem extract (Figure 4).

Stan's poem paints a bleak image that relays fears of becoming homeless and being abandoned; “he turned and walked away”. Stan noted “when I write about things like homelessness and things like that, it gives you an example of where I thought I'd end up because I was so bad with my nerves and my drinking” (Stan, first interview). It is difficult to engage with recovery processes when basic needs such as food and housing are not being met. One participant discussed the cost‐of‐living crisis as a barrier to accessing care; “I had to end my therapy because it was costing too much… cost of living now, for some people, it's a lot of money” (Cam, second interview). Cam's comments also highlight health inequalities in terms of a lack of access to mental health services, and as a result they utilised private therapy which was unaffordable. These experiences illustrate the impact deprivation can have on reducing physical safe spaces in an individual's daily life, creating barriers to recovery.

Intersectional stigma was a prominent experience for all participants relating to both SMI and other parts of identity including gender, sexuality, and disability. For example, Cam experiences a conflict between his religious and sexual identity: “I won't go to church because unless they start accepting people who are different, for their sexuality, I don't think I feel comfortable going to Mass or church.” Cam's experiences of stigma led to internalised homophobia that exacerbated his mental illness, “I have moments where I feel being gay is wrong, but it effects loads of gay people. It is the way society is built” (Cam, first interview). Such intersectional stigma adds additional barriers to coping with SMI as it reduces supportive social networks.

**Figure 5:**
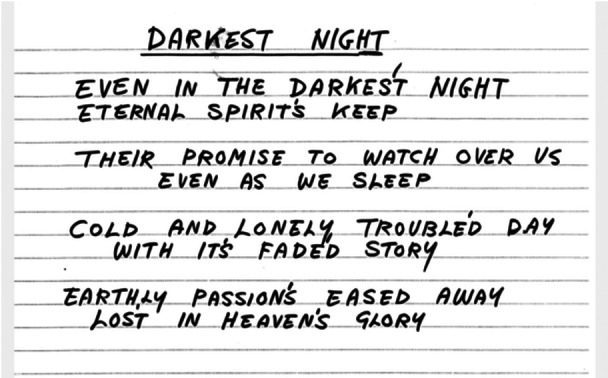
An excerpt from the Poem ‘Darkest Night’ by Stan.

Lastly, many of the participants demonstrated a desire to live well with SMI. A desire to live well manifested itself differently amongst the participants. For some it related to spirituality, illustrated by Stan's poem in Figure 5 written during a period of crisis, who notes that “It connects with the way that I was thinking, and even when I'm thinking now, even in the darkest night” (Stan, first interview).

The poem highlights an imperceptible desire and hope of recovery through the imagery of being ‘watched over’ by spirits. Stan found it hard to articulate the sensation commenting; “I knew there was something. You know? Healing, somewhere” (Stan, first interview). Stan's poetry provided a tool to express his desire to live well, however a personal interest in the arts in itself can be a desire to live well as articulated by Alex: “If you're intent is to get good at something…or enjoy something, you'll put [in] time and energy, you'll put yourself into it.” (Alex, realist interview). As such, opportunities to engage with creative activities within a community setting can appeal to an individual's desire to live well.

### 3.2 Developing the Creative Communities Framework

**Figure 6:**
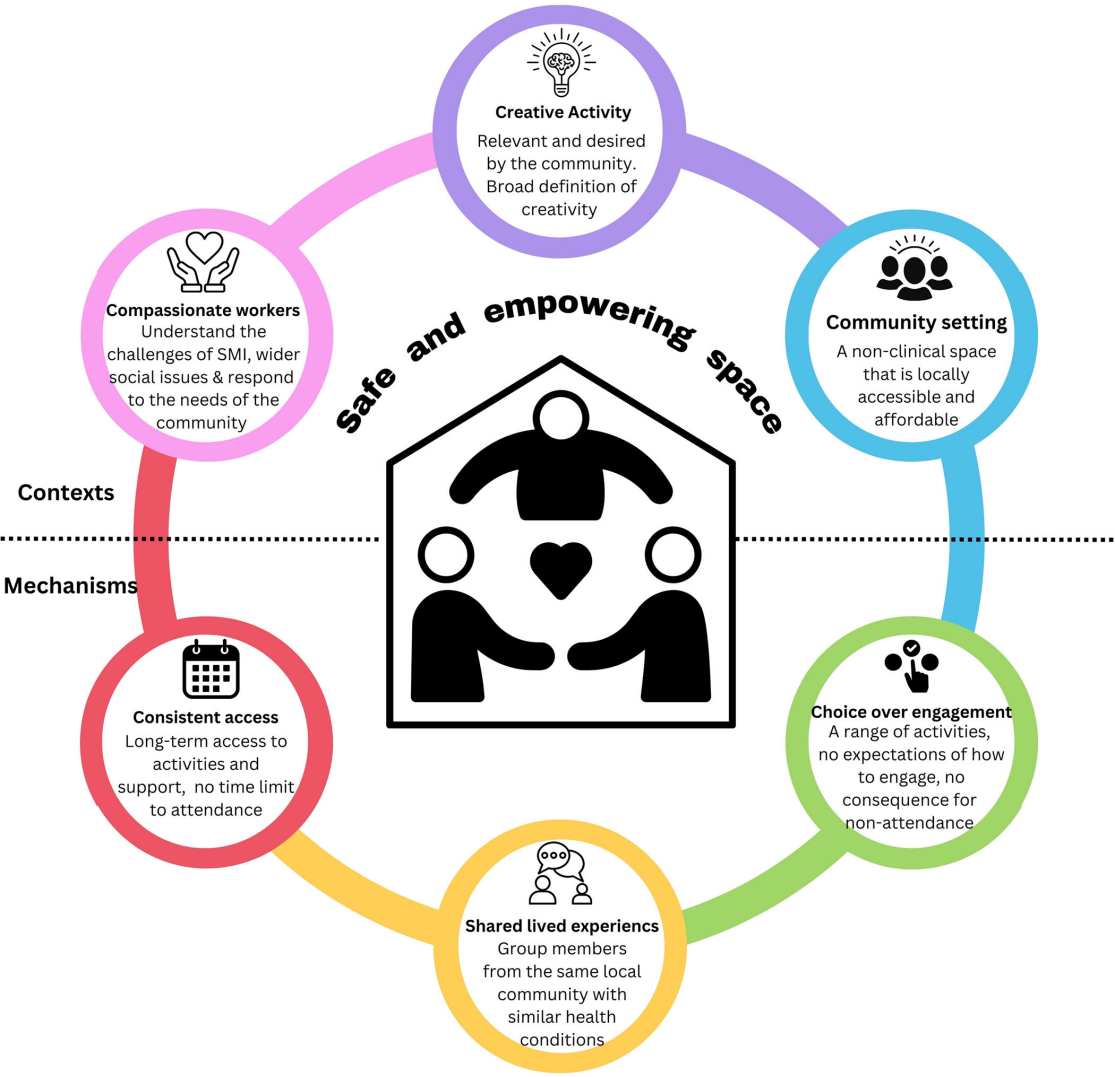
The creative communities framework.

The programme theory outlines six key ingredients, three contextual features of the intervention and three mechanisms, that operate within the SMI lived experience. These ingredients establish the Creative Communities Framework as they highlight factors that are important to establishing a safe and empowering space within the intervention. As such, the framework presents a practicable structure of how a safe intervention space can be created at both an organisational and individual level, outlined in Figure 6.

**Figure 7:**
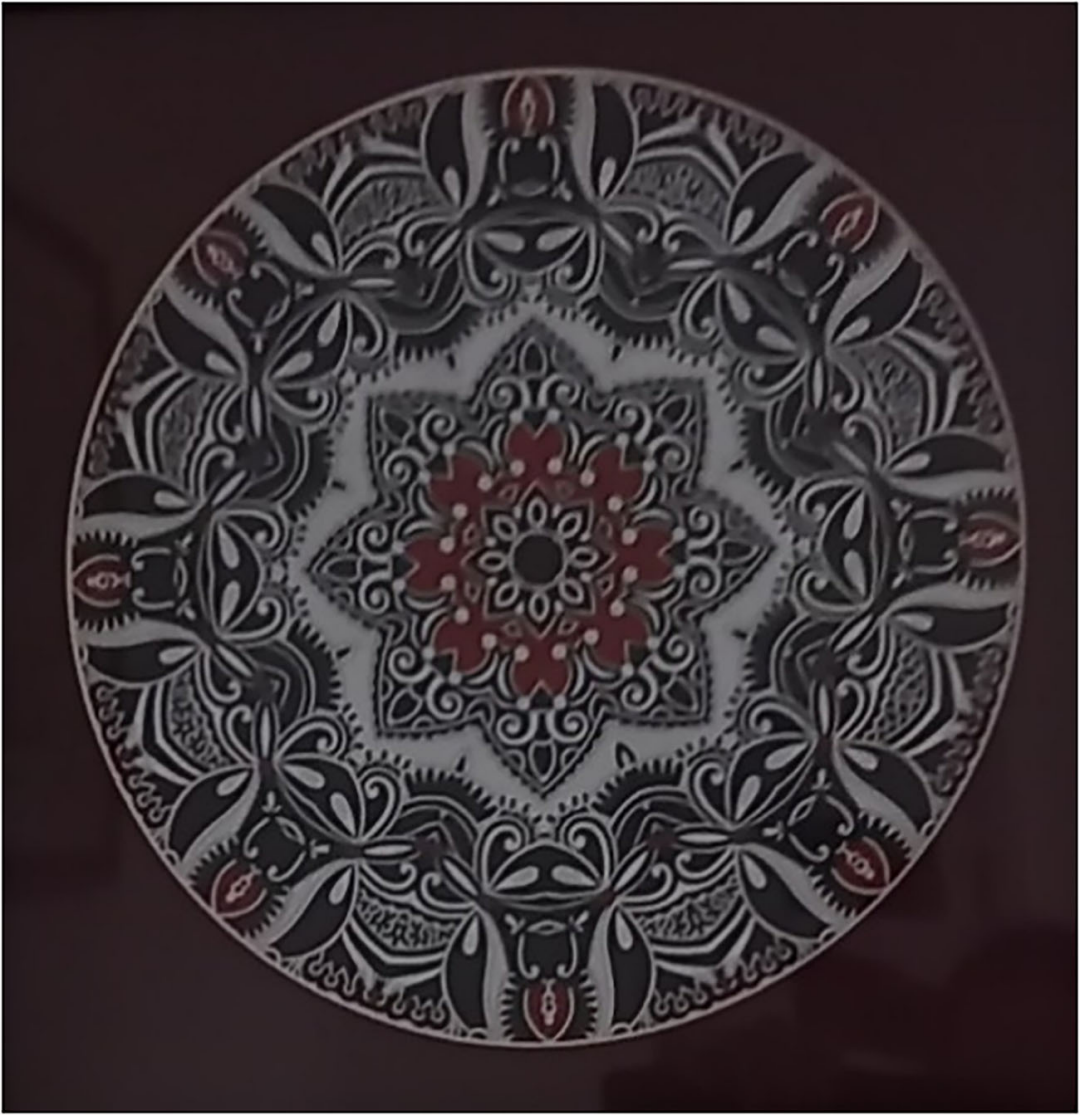
A printed mandala image, filled and modified by George with fine liner pen.

The intervention introduces three new contextual ingredients within the lived experience of SMI; (1) compassionate workers (including staff and volunteers); (2) creative activities; (3) a community setting (i.e., nonclinical). These three contextual ingredients appear in the top half of the Creative Communities Framework and offer an alternative context to that of living with SMI. Compassionate workers are key active agents within the intervention as they understand and respond to the needs of the local community, illustrating the ongoing and dynamic process of this ingredient: “We're constantly listening and learning and adapting with our community” (Liz, worker, realist interview). Compassion towards SMI presents an alternative experience to the stigma individuals face in other parts of their lives. The creative activity is equally important as it provides opportunities for experiences away from the daily challenges of SMI. This was exemplified by George who presented the artwork in Figure 7 in his interview stating: “Takes my mind off everything I think because I've got to concentrate… so the concentration just gives you a blank space, you know, which is good.”

When workers organise activities that are of interest to their local community, it can also appeal to an individual's desire to live well. Furthermore, this demonstrates the process of compassionate workers listening and responding to the needs of their community, exemplified by Viv's experience; “We've just done an 8‐week course on watercolours, which I asked for… I didn't go to school. I didn't do art.” (Viv, second interview). Viv's experience illustrates her interest in the arts and providing opportunities motivates her to engage. Plus, her comments highlight how community organisations are responding to the needs of their community, as well as societal inequalities, for example, equal access to the arts. An important feature of this access is the community setting, specifically that the activities physically take place within the community and are affordable. Local resources present an alternative to deprivation and health inequalities, such as inconsistent access of mental health services. The importance of a community setting is exemplified by June's experiences of accessing an arts activity within secondary care: “I started the jewellery making and it was only 1 day a week, but it meant travelling from [hospital]. The staff weren't terribly helpful, and I just had to give it up…” (June, first interview). June's reflections highlight that not being able to easily access an activity is a barrier to engagement. Furthermore, her experience also illustrates the importance of compassionate workers to facilitate engagement and how all three contextual ingredients need to be in place for intervention success.

When the intervention context is introduced within the lived experience of SMI, three mechanisms are activated; (1) choice over engagement fosters empowerment; (2) connect to others via shared experiences; (3) consistent access reinforces empowerment and connecting to others. The mechanism ingredients within the Creative Communities Framework appear on the bottom three sections of Figure 6. The mechanism ingredients provide an explanation of how individuals with SMI respond to the intervention context. It is these responses that develop the outcome of a safe and empowering creative community in which recovery can occur, as indicated in the centre of Figure 6. A safe and empowering space is created by allowing individuals to have alternative experiences to SMI as Dan explains:

“[group member] said the other week that… it was like going into a different world… It is that bubble, and it is not a bubble that is about protecting them, it is a bubble where things can happen that are different.”

(Dan, worker, realist interview)

Findings from the current research found that all three mechanisms also need to be activated to develop a safe and empowering creative community. Each mechanism has been outlined below with an example CMO configuration drawn from the data to illustrate how it is activated by both the intervention context and lived experience of SMI. The meaning of feeling safe and empowered is also articulated in each example. A programme theory is developed from multiple CMO configurations, however only three salient examples have been outlined below.

### 3.3 Mechanism One: Choice Over Engagement Fosters Empowerment

**Figure 8:**
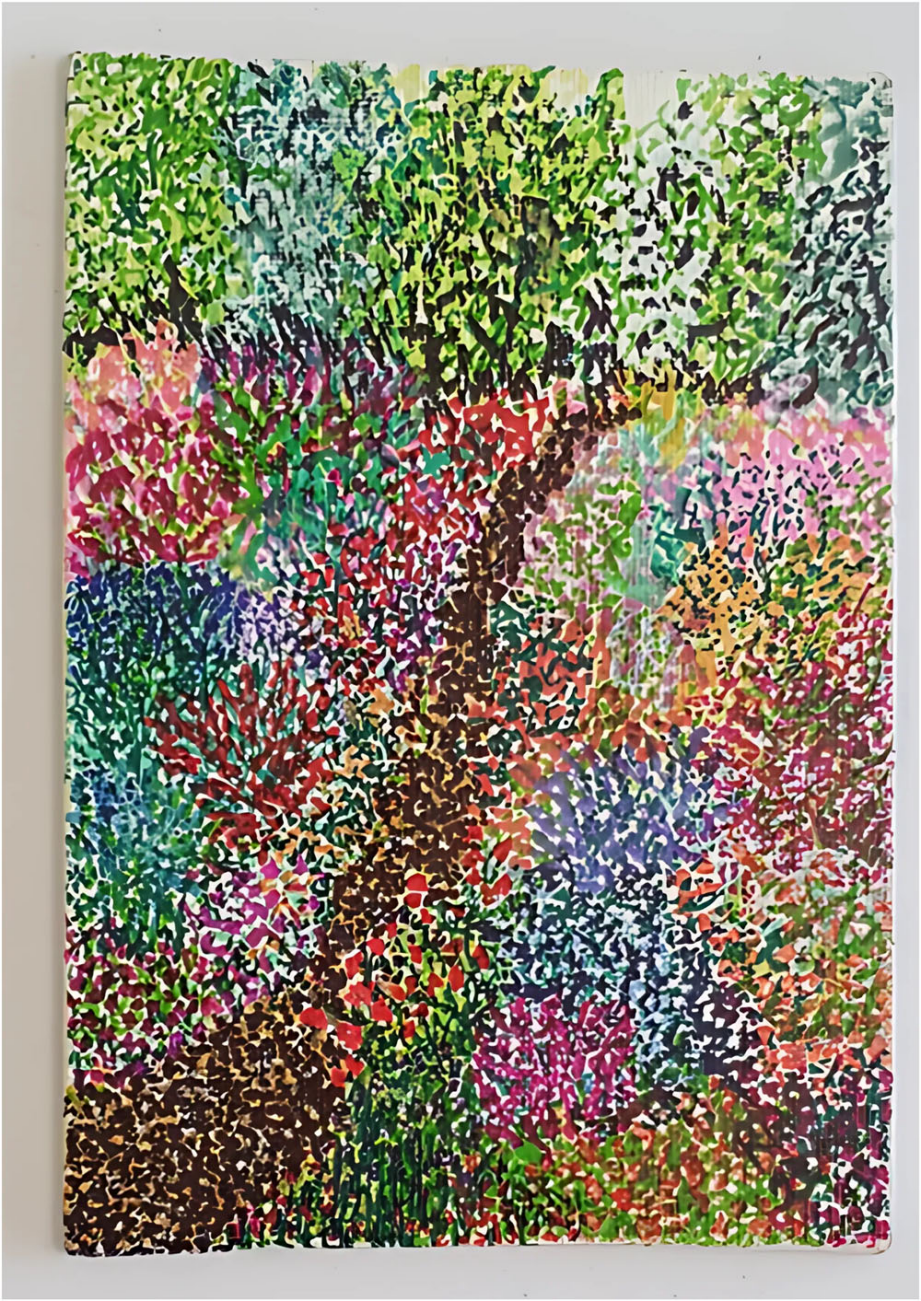
A dot work painting of a garden scene presented by participant June in her first arts elicitation interview.

Choice over engagement is activated when workers organise a range of activities and promote flexible engagement so individuals can take part on their own terms. Engagement can include how often individuals attend, level of participation and level of interactions with others, as worker Bev commented: “You are not forced into anything you don't want to do. It is not like, ‘oh the activity begins’, you know, it is very casual. That is part of why it works” (realist interview). Arts activities also provides an opportunity to focus on something other than SMI, removing expectations of recovery. June presented the painting in Figure 8 created using dot work, which is a time consuming and intricate process. In this way, arts practice gives June a choice over what her mind focuses on “I get totally lost and time just disappears, because it's just like zoning out” (June, first interview).

Individuals often respond to having choice by feeling empowered, particularly given the disempowering experiences of living with SMI and health inequalities. Paige, who lives with SMI and neurodiversity, relies on unhelpful coping strategies such as masking due to a fear of stigma. Within her community arts group however, she doesn't feel the pressure to behave in a certain way and so feels safe to be herself without masking, exemplified by the CMO configuration within the below quote:

“I don't really feel like I have to wear a mask [context of SMI] because I can just zone in on my art [intervention context] and not really speak to anyone if I don't want to speak to anyone… and if I want to speak to people, I can speak to people. If I don't want to do anything and I just want to sit there, yeah, like I can do… [mechanism: choice over engagement] I can really do my own thing and be my own person in the art groups, it's it's really, really good, I think [outcome].”

(Paige, first interview)

Paige's' expereinces highlights how she feels empowered within the art group as she does not have to conform to social expectations and interactions. This in turn creates a feeling of safety to be her ‘own person’, as she does not experience stigma within this space and does not feel the need to mask. These findings indicate that she is also engaging with the recovery processes of empowerment and identity within her creative community.

### 3.4 Mechanism Two: Connect to Others via Shared Experiences

Compassionate workers understand the need to treat individuals as people, not illnesses, as worker Liz notes; “You come in and you're a fresh person… I don't have a file to say that you've had schizophrenia and you've been in hospital for 5 years previous to this. I just know that you've come here and that you're interested in doing art” (realist interview). The compassionate approach fostered by workers and established within the groups provides an alternative to experiences of stigma. Individuals with SMI respond by connecting to others, sometimes developing friendships, which makes the space within the group feel safe. Workers are key active agents to maintaining a compassionate and inclusive social dynamic within the group as Bev explains:

“[group member] is from an Asian community… there may have been a little bit of, not unfriendliness, but she noticed it in the group, but she said that seems to have all gone now.

It is just about us, as facilitators of the group, making and creating a safe environment… I think if somebody's new coming into a group, you've got to be really welcoming… Now she is fully engaged, she's bringing biscuits in, she's chatting to everybody”

(Bev, realist interview)

Here Bev alludes to the potential for discrimination that could have removed feelings of safety for certain group members, yet through Bev's intervention this was addressed enabling the participant to connect to others.

The focus on the social space as an artistic one also provides a shared experience which connects people beyond SMI, exemplified by the below CMO configuration within a quote from Alex.

“[Art is] something that is outside of yourself that other people can identify with (intervention context). You can be incredibly vulnerable and leave it [in the art] (outcome). So, the catharsis is really strong, and that catharsis is what normally starts conversations between people (mechanism: connect to others) who have experience of not being heard, not knowing, feeling like they don't know who they are (context of SMI).

(Alex, realist interview)

The examples from both Bev and Alex highlight the different ways creative communities can allow people to connect and the important role both the compassionate workers and creative activity can play. Alex highlights that when you can connect to others, the space becomes one in which you can become ‘vulnerable’, indicating a feeling of safety. This mechanism also highlights an engagement with the connectedness recovery process, illustrating how creative communities enable individuals with SMI to live well.

### 3.5 Mechanism Three: Consistent Access Reinforces Empowerment and Connecting to Others

Consistent access is created by both compassionate workers and the community setting. Workers understand the long‐term needs of individuals with SMI and continue support regardless of engagement as exemplified by Liz “I make an effort to text everyone and say looking forward to seeing you on Wednesday. If someone doesn't come, I text them and say sorry, we didn't see you” (Liz, realist interview). Many participants commented on the importance of long‐term support to aid recovery: “I think the key issue is that the groups have to have continuity and consistency within them… short term goals might be really nice, but they are not very effective… in terms of people's mental health and recovery” (Sam, second interview). When individuals are given long‐term access, it reinforces the responses from mechanisms one and two, i.e., feeling empowered and developing positive relationships. Viv has found a constant source of support through her art group for over thirty years, particularly as the support they offer was within the community in which she lived. The workers were able to reach out to her directly to offer support, which empowered her to engage with the organisation and become part of their community. Viv's experiences are demonstrated in the below quote with a CMO configuration applied:

“I first started engaging with [charity] when I was about 15, 16… I was living away from home and I was living with a boyfriend and he was a graffiti artist at the time… We were sitting in the subway sketching… (context of SMI) Then [charity workers] started talking to us and came and gave us sketchbooks, showed us where their base was and resources… [intervention context]. Since then, I've always known about [charity] and the work they do. They are fantastic… I've been in and out of [charity] at different stages of my life when I've needed it… It is a vital resource for me, these sessions, these art classes [mechanism: consistent access]. Otherwise, my life would be on a downhill dip… allowing me to come to these sessions, is giving me a life [outcome]”.

(Viv, first interview)

Consistent access to her creative community has supported Viv to live well with continuing challenges of SMI over several decades. Such access stands in contrast to clinical care that is often time limited. Consistent access empowers Viv to access her group based on her own needs as she knows she will be welcomed, even after a period of absence. Such access contributes to the feeling of safety as individuals with SMI can rely on being accepted.

## 4 Discussion

The current paper presents findings from a realist evaluation to answer the specific question how, why and in what context do community organisations establish a safe and empowering space that allows individuals to engage with recovery processes? These findings represent one part of the full realist evaluation to detail the initial development of the Creative Communities Framework. The theory developed from this realist evaluation revealed six key ingredients which are needed to develop a safe and empowering space in which recovery can occur. These ingredients were used to develop the Creative Communities Framework to provide a practicable structure to illustrate how community‐based arts activities organised by compassionate workers (intervention contexts), allow individuals to have consistent access to opportunities to connect to others and feel empowered through choice (mechanisms). Whilst the interactions between contexts and mechanisms are dynamic and complex, the framework highlights features of the intervention that can be influenced by an organisation, for example, locating an intervention within a community setting. Therefore, the framework presents a guide to inform intervention development and delivery. The six ingredients develop a space in which alternative experiences can occur that contrast and counteract the negative lived experience of SMI. It is not to suggest however, that by simply having six ingredients to an intervention that a safe space will automatically be developed, and SMI recovery will consistently be engaged. The ingredients themselves represent processes that are dynamic and involve ongoing engagement from the people within the space. This was exemplified in the findings by Bev's experiences of having to continually maintain the safe space by managing interpersonal differences. In doing so however, a feeling of safety was established which provides alternative experiences that enables individuals with SMI to engage with personal recovery processes. Several recovery processes can already be identified within the six key ingredients. For example, mechanism two, connecting to others, reflects the connectedness recovery process identified by Leamy et al. (2011), and mechanism one demonstrates both the empowerment and identity recovery process (Leamy et al. 2011). These findings illustrate the complex and interlinked nature of engaging with personal recovery processes.

The six ingredients identified in this study can be seen as creating ‘recovery capital’ that increases the likelihood that an individual will engage with recovery. Recovery capital is a term used within addiction research, with increased recovery capital relating to more resources for individuals to draw upon to engage with recovery processes (Cano et al. 2017). Recovery capital can relate to personal, social and community resources, with community capital being identified as central to long‐term recovery within addiction (Cano et al. 2017). The ingredients identified in our study feature resources stemming from the individual (e.g., responses such as empowerment), social (e.g, compassionate workers facilitating connection to others) and community (e.g., locally based and accessible). The combination of these ingredients across different social layers could explain the resulting recovery capital, and why when one of these ingredients is removed, a safe and empowering space is not created. Rankin et al. (2009) explored clinical perspectives and found that healthcare workers, despite being compassionate towards individuals with SMI, are restricted by institutional policies and targets. Furthermore, clinical spaces tend to be physically distant from the area in which individuals with SMI live, resulting in inconsistent or time‐limited care (Rankin et al. 2009). Such research emphasises the need for multiple contextually based ingredients to be in place to increase recovery capital. The Creative Communities Framework identifies the specific ingredients needed within community arts, which has implications to inform the way community interventions using creative activities are designed and delivered. This study also suggests that arts activities appear to play a particular role within this process by providing opportunities for alternative creative experiences beyond SMI symptoms. In particular, the arts activities on offer appear to appeal to an individual's desire to live well and provides motivation to engage.

A programme theory is contextually specific explanation of how an intervention works (Jagosh et al. 2014; Pawson 2006; Pawson and Tilley 1997). As such, it is important in realist evaluation to conceptualise the findings at a higher level of abstraction to understand how the identified mechanisms may apply in different contexts (Astbury 2018). To this end, similarities can be drawn between the Creative Communities Framework and self‐determination theory which asserts that autonomy, competence, and relatedness are basic human needs (Deci and Ryan 2008). When these needs are fulfilled, an individual becomes self‐determined (i.e., asserts control over their life) which provides autonomous motivation for personal growth and is associated with improved health outcomes (Deci and Ryan 2008; Mancini 2008). Self‐determination is developed when these three basic needs are met and are linked with intrinsic goals, i.e., linked to personal development rather than external rewards (Deci and Ryan 2008). The Creative Communities Framework illustrates how self‐determination and autonomous motivation begin to develop through engaging with arts activities that are intrinsically important to the group members. For example, when individuals with SMI meet compassionate workers, they can begin to develop connections to others, which highlights how the basic need of relatedness is beginning to be met. A similar process is also seen within formal therapeutic settings (Brown and Parry 2022; Laugharne and Priebe 2006). Research has found that the therapeutic alliance has a greater impact on recovery than the type of therapy, highlighting the importance of developing positive relationships for individuals with SMI to become self‐determined (Harris 2004; Martin et al. 2000; Pearse et al. 2020; Smith et al. 2010). In addition, the feelings of empowerment identified within mechanism one aligns with the basic need for autonomy. Continued access to safe and empowering creative communities ensures repeated exposure to alternative experiences that over time enable individuals to become more self‐determined and engage in multiple personal recovery processes.

### 4.1 Strengths and Limitations

The Creative Communities Framework presents a structure to explain how community arts interventions develop safe spaces in which recovery from SMI can occur. The six ingredients identified within the framework offer researchers, facilitators and practitioners a guide to evaluate such interventions and inform programme development. However, detail of such application is not provided within this article as the current paper presents findings from the first part of the realist evaluation and the initial development of the Creative Communities Framework. Further details are also needed to explain the processes that are activated within a safe and empowering creative community and how recovery processes are engaged. For example, details of the role of the arts within such interventions, and how these interventions operate within other social systems are not provided within this paper. Given the complexity of the processes involved it was not possible to present the entire evaluation within one paper and initial insights into the processes within a safe and empowering creative community can be found within the realist systematic review (Peters et al. 2023). A potential limitation of realist research is that explanatory insights can become contextually based which limits how widely they can be utilised (Pawson 2006; Pawson et al. 2005). Future research should examine the utility of the framework in different contexts to assess the scope of its applicability.

## 5 Conclusion

The findings from this study identify six key ingredients to develop community‐based interventions that will increase the recovery capital for individuals with SMI, enabling engagement with recovery processes. Participation in community arts activities can be a valuable way for people with SMI to engage in recovery processes. Creative activities can address structural drivers of mental illness, and tap into fundamental components of wellbeing, including personal identity, which are often neglected in ‘traditional’ clinical interventions for SMI. As such, community arts can be a core component of addressing SMI in a holistic manner, and providing access to creative communities should be considered as an important part of care plans.

## Author Contributions

**Louisa Anne Smyllie‐Peters:** study conceptualisation, data curation, formal analysis, methodology development, project administration, resources and software set‐up, validation, visualisation and writing. **Tim Gomersall** and **Mike Lucock:** study conceptualisation, methodology, supervision, validation, visualisation and writing.

## Ethics Statement

Ethics Approval received from the University of Husserdield School Research Ethics and Integrity Committee. Reference: SREIC/2021/117.

## Consent

Informed consent was gained from all participants, including the anonymised use and publication of artwork images.

## Conflicts of Interest

The authors declare no conflicts of interest.

## Supporting information

Supporting Materials.

## Data Availability

The authors confirm they have full control of all sysnthesis details and that they agree to allow the journal to review this if requested.
