# Supplementary material for: Developing the Creative Communities Framework for Living Well With Serious Mental Illness: Findings From a Realist Evaluation
Source: J Community Psychol. 2025 Jul 10;53(5):e70028. doi: 10.1002/jcop.70028 (PMC12242901; doi:10.1002/jcop.70028)
Supplement: Supplementary file 1 — Supporting Materials. [file JCOP-53-0-s001.docx]

**Supplementary Materials**

**An outline of the template versions developed during the evaluation**

A priori codes

| C | Context of living with SMI |
| --- | --- |
| C1 | - Feel isolated |
| C2 | - Feel stuck |
| C3 | - Have a negative illness identity |
| C4 | - Experience stigma |
| C5 | - - Poor experience within mental health services |
| C6 | - Challenges coping with illness |
| C8 | - Challenges with self-expression |
| CI | Intervention context |
| CI1 | - Feels safe |
| CI2 | - Empowering |
| CI3 | - - Arts focused but for mental health |
| CI4 | - - Community-based (none-clinical) |
| CI5 | - - Voluntary |
| M1 | Mechanism 1: Learning to cope with illness (resource) develops a sense of control over illness (reaction) |
| M1.1 | - Learning artistic expression (resource) developing coping strategies (reaction) through |
| M1.2 | - Escapism (reaction) |
| M1.3 | - Gaining personal insight (reaction) |
| M1.4 | - Therapeutic processing (reaction) |
| M1.5 | - Using art to cope (resource) develops sense of control over illness (reaction) |
| M2 | Mechanism 2: Connecting with others (resource) makes you feel accepted (reaction) through |
| M2.1 | - A shared experience (resource) develops connection with others (reaction) |
| M2.2 | - Valued interactions (resource) validating self (reaction) |
| M2.3 | - gaining social support (resource) helps cope with illness (reaction) |
| M3 | Mechanism 3: Overcoming challenges (resource) develops self-awareness (reaction) |
| M3.1 | - learning to cope through art (resource) and be confronting & uncomfortable (reaction) |
| M3.2 | - feeling safe (resource) encourages risk-taking (reaction) |
| M3.3 | - learning artistic expression (resource) enables self-reflection on evidence (reaction) |
| M4 | Mechanism 4: Coping/connectedness/overcoming challenges (resources) develops self-awareness of abilities/strengths/hope/positives (reactions) |
| M4.1 | - Evidence of ability (resource) promotes self-reflection (reaction) |
| O | Outcome: to redefine your identity beyond illness, helping to move towards acceptance of illness into identity (O). |
| O1 | - (O) redefine self beyond illness |
| O2 | - (O) accept illness into identity |
| O3 | - - (O) illness perception changes from overwhelming to manageable |

**Initial Template**

| C1 Negative experiences of living with SMI  C1.1 Experience intersectional stigma  C1.2 Negative side effects of clinical care  C1.3 Challenges of coping  C1.3.1 All the time  C1.3.2 Lack of adaptive coping strategies  C1.3.3 Compounded by comorbidities or multimorbidity's  C1.4 Other hardships in life  C2 Leads to barriers of SMI recovery  C2.1 Personal world is small-restricted through  C2.1.1 Isolated  C2.1.2 Feel lonely  C2.2.2 Feel stuck  C2.2 Negative impact on identity  C2.2.1 Lost or taken  C2.2.2 Broken  C2.2.3 Hidden  C2.2.4 Overwhelmed  C2.3 Challenges of self-expression  C2.4 Fear  IC Intervention context  IC1 Feeling of safety through  IC1.1 Place - community-based  IC1.2 Activity  IC1.3 Group support  IC2 Empowering through  IC2.1 Choice  IC2.2 Equality - No sigma or hierarchy  IC2.3 No expectations but opportunities  IC3 Arts focus, but designed for SMI group  M1 Learning to cope with illness develops a sense of control over illness  M1.1 Self-expression from learning art  M1.2 Communication from learning art  M1.3 Coping from learning art  M1.3.1 Escapism  M1.3.2 Gaining insight - personal and societal  M1.3.3 Therapeutic processing | M2 Connecting with others makes you feel accepted through  M2.1 Shared experience  M2.1.1 SMI  M2.1.2 Arts  M2.1.3 No stigma  M2.2 Valued interactions  M2.2.1 Self  M2.2.2 Art  M2.2.3 No stigma  M2.3 Connect and collaborate with others  M2.4 Validate self  M3 Challenges of creative recovery  M3.1 learning to cope through art and be confronting & uncomfortable  M3.2 social dynamics can be challenging  M3.3 learning new art skills can be hard  M3.4 feeling safe, joy and gaining social support provides motivation to move through challenges and take risks  M4 Experience positive emotions joy through  M4.1 Act of creativity and final product  M4.2 Coping  M4.3 Talking to others  M5 Coping-connectedness-overcoming challenges develops self-awareness of abilities-strengths-hope-positives  O1 Redefine self beyond SMI through  O1.1 Reconnecting with lost self  O1.2 New roles in group  O1.3 Gaining self-confidence  O2 Accept illness into identity when with the group  O3 Ripple effects of  O3.1 Helping others  O3.2 Social change  O3.3 Improve relationship with family  O3.4 Regain or seek employment  O3.5 Adjust coping strategies outside group |
| --- | --- |

**Version 1**

| **PT1 Creating a safe and empowering context**  C1 Negative experiences of living with SMI  C1.1 Social and health inequalities  C1.2 Experience intersectional stigma (macro-meso)  C1.3 Some degree of negative side effects of clinical care (meso-micro)  C1.4 Challenges of coping (micro)  C1.4.1 All the time  C1.4.2 Lack of adaptive coping strategies  C1.4.3 Compounded by comorbidities or multimorbidity's  C1.4.4 Symptom severity  C2 Leads to barriers of SMI recovery  C2.1 Imposed loneliness  C2.2 Negative impact on identity  C2.2.4 Overwhelmed  C2.2.5 Imposed SMI ID  C2.3 Challenges of self-expression  C2.4 Fear and no hope  M1 Choice  M2 Equality  M3 Opportunities over expectations  M4 Focus on creativity but for people with SMI  M5 Consistency  M6 Shared experience  Developing a safe and empowering space for self  Feel safe  Safe space for self  Empowered  **PT2 Engaging with the identity change recovery process**  C Living with SMI & attending a safe and empowering space for self  M1 Learning to cope with illness develops a sense of control over illness  M1.1 Self-expression from learning art  M1.2 Art is acceptable communication tool  M1.3 Coping from learning art  M1.3.1 Escapism  M1.3.2 Gaining insight - personal and societal  M1.3.3 Therapeutic processing  M1.3.4 Symptom management | M2 Connecting with others makes you feel accepted through  M2.1 Shared experience  M 2.1.3 Other parts of ID  M2.1.1 Living with SMI  M2.1.2 Arts  M2.2 Valued interactions  M2.2.1 Self  M2.2.2 Art  M2.2.3 No stigma  M2.3 Connect and collaborate with others  M2.4 Validate self  M3 Challenges of creative recovery  M3.1 learning to cope through art can be uncomfortable  M3.2 social dynamics can be challenging  M3.3 learning new art skills can be hard  M3.4 feeling safe, joy and gaining social support provides motivation to move through challenges and take risks  M4 Experience positive emotions and hope through  M4.1 Act of creativity and final product  M4.2 Control over illness through coping  M4.3 Connecting to others  M5 Coping-connectedness-overcoming challenges develops self-awareness of SMI-abilities-strengths-hope-positives  O1 Accept illness into identity to some degree - over time  O2 Redefine self beyond SMI through  **PT3 Ripple effects of positive identity change**  C1 Living with SMI but defining self beyond SMI through attending a CBAA  C2 Reconnecting with lost self  M1 Self-awareness  M2 Gaining self-confidence  M3 Valued voice  M-O Sense of purpose  O1.2 New roles in group  O3 Ripple effects of  O3.1 Helping others  O3.2 Social change  O3.3 Improve relationship with family  O3.4 Regain or seek employment  O3.5 Adjust coping strategies outside group  O3.6 Service development  O3.7 Reduce symptoms |
| --- | --- |

**Version 2**

| **PT1 Creating a safe and empowering context**  C1 Challenges of coping with fluctuating severity of serious mental illness  C1.1 All the time  C1.2 Lack of adaptive coping strategies  C1.3 Compounded by comorbidities  C1.4 Fluctuating symptom severity  C1.5 Challenges of self-expression  C2 Feels isolating, overwhelming, and hopeless  C3 Exacerbated by social and health inequalities  C3.1 Intersectional stigma  C3.2 Deprivation  C3.3 Let down by services  C4 A need (whether internal or external) to engage with recovery  C4.1 External e.g., family dependants, a positive relationship, desire for purpose  C4.2 Internal ego, Desire to feel like myself, denial of illness, interest in activity.  M1 Arts organisation reacts to the needs of the community  M1.1 Acknowledging this context  M1.2 delivering desired activities in areas with higher social and health inequalities  M2 Choice and empowerment  M2.1 range of accessible opportunities that promote creativity not recovery  M2.2 control over what activity they do and how they take part  M3 Valued interactions develops positive relationships  M3.1 treated like a person  M3.1.1 experienced charity workers who are supportive and understanding  M3.2 develop positive relationships  M3.2.1 through trust and a feeling of acceptance  M4 Consistency reinforces of empowerment & positive relationships  M4.1 long-term access  M4.2 continued support  O1 Developing a safe space  O1.1 Away from life’s challenges  O1.2 where is feels safe to be yourself  **PT2 Engaging with the identity change recovery process**  C Living with SMI & attending a safe and empowering space for self  M1 Learning arts skills aids self-expression and coping with illness through  M1.1 Art as an acceptable communication tool  M1.2 Temporary escapism  M1.3 Gaining insight - personal and societal  M1.4 Symptom management | M2 Connecting with others makes you feel accepted through  M2.1 Shared experience  M 2.1.3 Other parts of ID  M2.1.1 Living with SMI  M2.1.2 Arts  M2.2 Valued interactions  M2.3 Connect to others and gain social support  M2.4 Validate self  M3 Experience positive emotions and hope through  M3.1 Act of creativity and final product  M3.2 Control over illness through coping  M3.3 Connecting to others  M4 Challenges of creative recovery  M4.1 learning to cope through art can be uncomfortable  M4.2 social dynamics can be challenging  M4.3 learning new art skills can be hard  M4.4 feeling safe, joy, coping and gaining social support provides motivation to move through challenges and take risks  M4.5 Continue to live with SMI & comorbidity  M5 Coping-connectedness-overcoming challenges-positive emotions develops self-awareness of SMI-abilities-strengths-hope-positives  O1 Acceptance of illness into identity shows engagement in recovery processes  O2 Redefine self beyond SMI through reconnecting with self  **PT3 Ripple effects of positive identity change**  C1 Living with SMI but defining self beyond SMI through attending a CBAA  C2 Reconnecting with lost self  C3 Social identity gained through role in group  M1 Self-awareness  M2 Gaining self-confidence  M3 Valued voice  M4 Creating outside of group  M5 Sense of purpose  O1 Individual benefits  O1.1 Quality of life  O1.2Adjust coping strategies outside group  O1.3 SMI self-management  O2 Interpersonal ripple effects  O2.1 Helping others  O2.3 Improve relationship with family  O3 Societal contributions  O3.1 Social change  O3.2 Regain or seek employment  O3.3 Service development |
| --- | --- |

**Version 3**

| **PT1 Creating a safe and empowering context**  C1 Living well with serious mental illness is challenging when continuously coping with fluctuating symptom severity  C2 resulting in feelings of isolation, fear and being overwhelmed.  C3 Challenges to living well are exacerbated by  C3.1 experiences of intersectional stigma  C3.2 health inequalities  C3.3 Compounded by comorbidities  C3.4 reliance on maladaptive coping strategies  C3.5 Challenges of self-expression  C4. Despite these challenges and negative experiences there is a desire or need to live well with SMI.  M1 Addressing community needs through  M1.1 experienced workers who  M1.1. understand the context of living with SMI  M1.2 have the autonomy react directly in response to the needs of the community  M2 Empowering engagement through  M2.1 organising desired and accessible activities  M2.1.1 focus on creativity over recovery  M2.2 providing choice (and control over engagement)  M3 Developing positive relationships  M3.1 person-centred interactions  M3.2 promoting equality  M4 Consistency reinforces of empowerment & positive relationships  M4.1 long-term access  M4.2 continued support  O1 Establishing an alternative community  O1.1 counteract the challenges and negative experiences of living with SMI  O1.1.1 Positive relationships counteracts feelings of isolation  O1.1.2 person-centred interactions oppose experiences of intersectional stigma  O1.1.3 local and accessible activities counter the health inequality of deprivation  O1.1.4 consistency of access opposes the short-term nature of services  O2 where individuals feel safe to be themselves  **PT2 Engaging with the identity change recovery process**  C Living with SMI & attending a safe and empowering space for self  M1 Learning arts skills aids self-expression and coping with illness through  M1.1 Art as an acceptable communication tool  M1.2 Temporary escapism  M1.3 Gaining insight - personal and societal  M1.4 Symptom management | M2 Connecting with others makes you feel accepted through  M2.1 Shared experience  M 2.1.3 Other parts of ID  M2.1.1 Living with SMI  M2.1.2 Arts  M2.2 Valued interactions  M2.3 Connect to others and gain social support  M2.4 Validate self  M3 Experience positive emotions and hope through  M3.1 Act of creativity and final product  M3.2 Control over illness through coping  M3.3 Connecting to others  M4 Challenges of creative recovery  M4.1 learning to cope through art can be uncomfortable  M4.2 social dynamics can be challenging  M4.3 learning new art skills can be hard  M4.4 feeling safe, joy, coping and gaining social support provides motivation to move through challenges and take risks  M4.5 Continue to live with SMI & comorbidity  M5 Coping-connectedness-overcoming challenges-positive emotions develops self-awareness of SMI-abilities-strengths-hope-positives  O1 Reinforces the context of community development  O2 Redefine self beyond SMI through  O2.1 Reconnecting with self  O2.2 Gaining social roles  O3 Acceptance of illness identity  **PT3 Ripple effects of positive identity change (Icing on the cake P54)**  C1 Engaging in recovery processes  C1.1 identity change  C1.2 Connectedness  C2 Yet challenges of SMI and social and health inequalities continue.  M1 Sense of control in life through  M1.1 Self-confidence  M1.2 Problem solving  M1.3 Self-awareness  M2 Feel worthwhile through having a sense of purpose  M3 Coping strategies supports SMI self-management  O1 Maintaining recovery - living well with SMI  O2 Societal contributions  O2.1 Helping others  O2.2 service delivery  O2.3 Social change  O2.4 Seeking employment or education  O2.5 Feeds into maintaining recovery |
| --- | --- |

**Final template**

| **Overarching Context: Lived experience of SMI**  C1 Living well with serious mental illness is challenging when continuously coping with fluctuating symptom severity   - C1.1 Continuous - C1.2 Compounded by other conditions - C1.3 Fluctuating symptom severity - C1.4 Feelings of isolation, fear and being overwhelmed.   C2 relying on unhelpful coping strategies   - C2.1 Challenges of self-expression   C3 Exacerbated by social and health inequalities   - C3.1 experiences of intersectional stigma   - C3.1.1 SMI   - C3.1.2 Disablism   - C3.1.3 Gender and sexuality - C3.2 health inequalities   - C3.2.1 Financial hardship   - C3.2.2 Poor access to services     - C3.2.2.1 Restrictions imposed by bureaucracy     - C3.2.2.2 Institutional discrimination - C3.3 Compounded by other conditions   C4 A desire to live well with SMI  **PT1 Establishing a safe and empowering context**  C1 Intervention context   - C1.1 Locally based and non-clinical - C1.2 Experienced workers - C1.3 Desired activities that focus on arts   M1 Feeling empowered through choice over engagement  M2 Person centred interactions develops positive relationships  M3 Consistency reinforces empowerment and positive relationships  O1 Establishing an alternative community  O2 Individuals feel safe to be themselves | **PT2 Engaging with the identity change recovery process**  M1 Learning coping strategies develops control over SMI   - M1.1 Art as a safe communication tool - M1.2 Escapism - M1.3 Therapeutic processing   M2 Achieving acceptance through connectedness   - M2.1 Shared experience   - M2.1.1 SMI   - M2.1.2 Arts   - M2.1.3 Other parts of identity - M2.2 Valued interactions - M2.3 Validate self - M2.4 Connect to others and gain social support   M3 Overcoming personal challenges   - M3.1 learning new creative coping strategies - M3.2 social dynamics can be challenging - M3.3 Continue to live with SMI - M3.4 feeling safe, joy, coping and gaining social support helps provides motivation to overcome challenges   M4 Positive emotions aid coping, connectedness and overcoming challenges   - M4.1 Creative practice - M4.2 Connecting to others   M5 Coping-connectedness-overcoming challenges-positive emotions develops self-awareness of SMI-abilities-strengths-hope-positives  O1 Reinforces the context of community development  O2 Positive self-awareness facilitate identity change  O3 Redefine self beyond SMI through   - O3.1 Reconnecting with self - O3.2 Gaining social roles - O3.3 Acceptance of illness identity   **PT3 Impact of living well with SMI**  C1 Engaging recovery processes including (but not limited to) identity change   - C1.1 Challenges of SMI continue (overarching context) - C1.2 Continue to access art activity   M1 Self-confidence provides motivation  M2 Feeling worthwhile from a sense of purpose  M3 Coping strategies supports SMI self-management  O1 Maintaining recovery (living well with SMI)  O2 Actively engaging in personal growth   - O2.1 Education - O2.2 Engaging in other activities   O3 Contributing to society   - O3.1 Helping others - O3.2 Service delivery - O3.3 Social change - O3.4 Employment |
| --- | --- |

**Example visuo-textual analysis of two artwork provided by June during their first interview.**


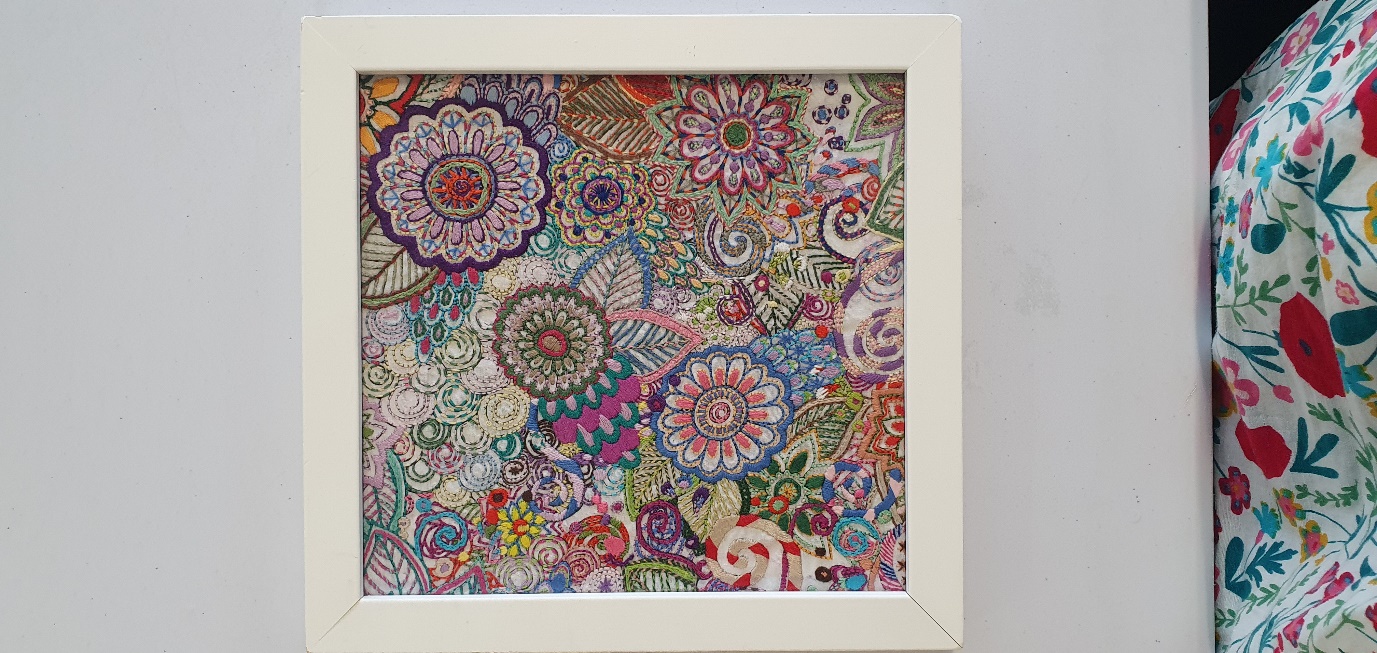

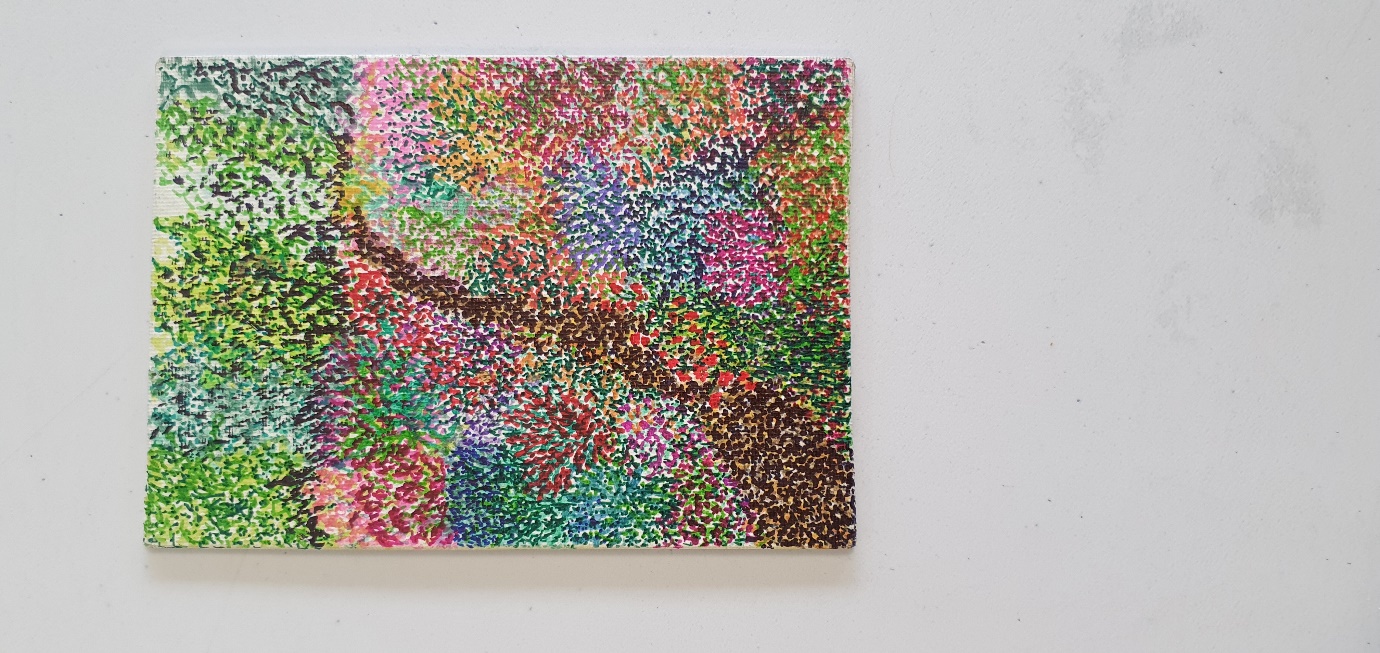


|  | **Artwork - drawing** | **Transcript quotes** | **Themes** |
| --- | --- | --- | --- |
| 1. **Description** | General notes   - Nature, flowers, landscapes - Figurative - Highly detailed - Colourful, bright, vibrant - Intricate patterns = movement - Busy - No pre-planning or pattern used - Requires high focus (both the make and look at)   Painting   - Path through a garden - Summer - Flower beds   Embroidery   - Multiple thread types - Highly textured | 1. (drawing) I think that's one I erm, one that I got a prize for that one. I got a prize. 'cause, it's sort of. It's you've got to tune into it. 2. it just has to materialize. OK, that's cool and I like that. I think I like colour, don't I? 3. (Interviewer: They’re very good) I never think, they are. 4. I do get a flowery phase, a bird phase, and a butterfly phase 5. It's a thing I used to do. A lot, and I don't now Know yeah, yeah, I can understand that. Because for a long time we had a nice garden. And yeah. We just, we had a dog, so we always went for walks, and you see nature in different seasons, but we sort of lost touch from that really. 6. Unfortunately, I get totally lost and time just disappears, and I get get meals.I don't get to bed till like 2:00 o'clock and. You can just keep going and going and going. Because it just. Started zoning how the television will still be on and I'll be zoned out somewhere 7. Colours, shapes, patterns keeps me happy really. 8. I think that's why, but partly why I like nature 'cause it's not. You know you can have. A slight fault flaw in flowers. 9. I had this funny feeling if. You paint a person. It mattered if it came out horrible. You wouldn't want to miss... If do somebody's picture and oh, that's awful. 10. (on embroidery)I think my favourite of all time is my embroidery that [inaudible] frame 11. So you start out with just vague shapes. And yeah. They do whatever you want to do with the stitches. And then it sort of, and it just comes out of it all right. So you choose your own colours, you choose your own stitches to. 12. I think it's just the fact of. I like this colours, variation of stitches and just pleasing shapes. I love this little bit here, 13. (On embroidery) I used to do when I was really little. My mum used to buy me. This fabric called binka. And it's like even weave. mini, but bigger. And I just used to. She'd buy me some threads in this fabric, and I'd just do patterns across it. |  |
| 1. **Interpretation** | - Love of nature - Organic, freedom - Draws you in, engrossing - Positive emotions | 1. ‘tune in’ engrossing 2. Enjoyment 3. Challenges of artistic judgement 4. Love of nature 5. Love of nature, happy memory 6. Engrossed 7. Enjoyment 8. Feel safe in nature 9. Challenges of artistic/social judgement 10. Proud of achievement 11. Organic process, mindful 12. Enjoyment 13. Happy memory from childhood | **Reconnecting with self**  Using arts to connect with past positive memories, in particular nature.  **Positive emotions overcome challenges**  Challenges of making and social judgement seem to be overcome by positive emotions from creating.  **Escapism**  Creating art works is used as a coping strategy to cope with SMI. |

**An example Qualitative Longitudinal Analysis across Viv’s interviews and experiences, followed by the resulting case summary**

|  | 1. **Living with SMI** | 1. **feeling safe** | 1. **Art / groups** | 1. **Experience of services** | 1. **Perception/feelings towards self** |
| --- | --- | --- | --- | --- | --- |
| 1. **What remained constant through time?** | Living with SMI & comorbidities are the same “I'm numb and it is all raging around me.” T1 | Wanting to isolate self - feels safe T1 | Access to community art group |  |  |
| 1. **What changes over time and in what way?** | Struggled to get a clear diagnosis over time, has received difference diagnosis labels. T1 | Realise isolation is only ok for a short time T1 | Seen the charity change over the decades. Used to have a bigger resource centre but offered less activities. Charity has diversified.  Another art group opened up to everyone after the pandemic and Viv disengaged due to too many unfamiliar people making her feel unsafe (T2)  During the pandemic her main community arts organisation kept in touch with her and sent her art packs for support, but she found it hard to engage on her own. (T2) | Struggled to get a clear diagnosis over time, has received difference diagnosis labels. T1 | Has become self-aware of own needs and behaviours over time. T1 |
| 1. **What changes (or lack thereof) are interrelated?** | 1a & 2e.  6a & 2e | 1b & 1c |  | Both SMI and learning difficulties. a1 + 5d |  |
| 1. **What are the mechanisms of change?** |  | Workers actively developing relationships (T1) | Choice over engagement. Viv requested to learn about watercolours and the charity organised a course on this topic (T2) |  |  |
| 1. **What contextual factors affect change?** |  | Long term access to her main art group for 30 years. (T1) | Staff members spent time getting to know her, which encouraged engagement with the charity (T1) | Poor experiences of stigma within health service is a barrier to receiving support from the healthcare system. Patronising. “he gave me a diagnosis I've never heard which sounded like he had made it up” (T1) |  |
| 1. **How is change, or lack of, experienced by the participant?** | Accepts who she is. It’s not about change, it’s about acceptance (T2) | Change to a community art group resulted in her feeling safe and disengaging. One group broadened their remit beyond specific conditions. |  |  | Has accepted her SMI and learning disability over the decades. Feels more able to be self within safe spaces such as community arts group. |

**Case Summary**

Viv lives with a number of life-long conditions including learning disability, autism, chronic pain, and respiratory issues, as well as living with PTSD and depression for most of her life. She left home when she was young and did not access education, but she always enjoyed art. As a teenager she created art on the street with her boyfriend at the time, finding free materials in everyday life. A local arts organisation was set up in her area with the aim to bring free arts access to areas of deprivation. Workers saw Viv making art on the street and gave her free materials to encourage her, and began to establish a relationship with her. Over time, she started to attend the charity office to make art and has attended the organisation throughout the past thirty years.

During the Covid-19 pandemic her community art group-maintained contact with Viv and sent her art materials to be able to create art at home. However, Viv found it difficult to engage with the activities when she was not able to go out and have the support of the group. Once restrictions were lifted, she started to attend her groups again. One group that she used to attend was aimed at individuals living with autism, but after the pandemic the organisation had diversified and opened the group to anyone. Unfortunately, this change in group dynamic meant that Viv felt unsafe with new people (strangers) attending and a new group facilitator. This meant that Viv disengaged from this particular group, however she remained at her arts for mental health group that has supported her over the decades.

Viv has come to accept her conditions over time and that they are life-long conditions that are part of who she is. She feels that she does not have to hide who she is in the community art space and often likes to sit quietly to make art, rather than talking to people. Creating artwork can sometimes help Viv to process thoughts and feelings, and having regular access to the group gives her something to do and gets her out of bed. However, Viv acknowledges that she will always have to manage her symptoms and feels that mental health services are dismissive of her experiences. She has regular support from personal carers and lives as well as she can but feels she cannot rely on mental health services.
